# Text-Book of Nursing

**Published:** 1886-01

**Authors:** 


A TEXT-BOOK OF NURSING, FOR THE USE OF TRAINING SCHOOLS, FAMILIES, AND PRIVATE STUDENTS. *Compiled by* CLARA S. WEEKS, *Graduate New York Hospital Training-School, Superintendent of the Training-School for Nurses, at Paterson, New Jersey. New York:* D. APPLETON & CO. 1885. *pp.* 396. *Chicago:* JANSEN, MCCLURG & CO.

Obviously a treatise on nursing should be a condensed and practical hand-book, yet comprehensive and thorough in its instructions, if it aspires to the position of a text-book.

All of these merits belong to this work in a high degree. It not only contains careful and sensible directions for almost all the duties pertaining to the management of the sick-room, but it is also furnished with a complete series of questions for review and examination, so that it is admirably adapted for use in training schools.

Private students and families will find the book of use, and practitioners, as well as their assistants, will receive some benefit from perusal of its pages.

Some of the chapters are headed as follows: Beds and Bed-making—Bed Sores; Circulation—Pulse—Temperature—Respiration—Ventilation—Warmth; The Observation of Symptoms, Medicines and Their Administration; Food and Its Administration; Enemata—Suppositories—Douches-Counter-Irritants—Cups—Leeches—Poultices—Fomentations, *etc;* Baths—Massage—Urine; Surgical Nursing—Operations—Bones—Fractures—Dislocations; Bandaging; Hemorrhages; Obstetrics; Emergencies; Sick Children—Questions for Review and Examination. E. W. A.

